# Diagnostic CT organ dose estimation in whole-body PET/CT examinations–comparison of a patient-specific monte carlo approach and a computational phantom-based CT dosimetry tool

**DOI:** 10.1186/s40658-025-00811-x

**Published:** 2025-11-26

**Authors:** Gwenny Verfaillie, Yves D’Asseler, Klaus Bacher

**Affiliations:** 1 Department of Human Structure and Repair, Ghent University, Ghent, Belgium; 2 Department of Nuclear Medicine, Ghent University Hospital, Ghent, Belgium; 3 Department of Diagnostic Sciences, Ghent University, Ghent, Belgium

**Keywords:** Nuclear medicine, Computed tomography (CT), Dosimetry, Monte carlo, Patient-specific dosimetry, Organ dose

## Abstract

**Background:**

Studies evaluating the impact of advances in CT dosimetry tools on CT organ dose estimations are often limited to a comparison with TLD measurements in anthropomorphic phantoms or a comparison of different dosimetry tools using computational phantoms and CT examinations performed at radiology departments. This study evaluates organ dose estimations obtained using a patient-specific Monte Carlo simulation and a computational phantom-based dosimetry tool for whole-body PET/CT examinations. In addition, the correlation of organ doses with the size-specific dose estimate (SSDE) was investigated.

**Methods:**

Using the Monte Carlo software ImpactMC, patient-specific organ doses were simulated in 100 adult patients using whole-body CT scans acquired on a Siemens Biograph mCT Flow and a GE Discovery MI PET/CT. For each patient, organ doses were also estimated using the computational phantom-based dosimetry tool NCICT. Absolute and normalised to CTDI_vol_ organ doses and percentage dose differences were assessed for CT acquisitions performed with tube current modulation (TCM). Statistical and regression analysis was performed to evaluate dose differences, their correlation with patient characteristics and the relationship with SSDE.

**Results:**

The average percentage difference of NCICT to ImpactMC organ doses across all organs and BMI categories for whole-body examinations performed with TCM was − 5% and − 22% for the Siemens and GE PET/CT, respectively. Strong variations are observed between patients. Depending on the organ of interest, NCICT under-or overestimates the organ dose. Nevertheless, depending on the PET/CT system, moderate to excellent agreement was found between organ doses estimated with NCICT and ImpactMC. No correlations were observed between the obtained organ dose differences and patient length (R^2^ < 0.1), while weak to no or moderate correlations were found with patient weight (0.2 < R^2^ < 0.6) and BMI (0.2 < R^2^ < 0.7). Very strong correlations (R^2^ > 0.9) were observed between the estimated organ doses and SSDE.

**Conclusion:**

Compared to the patient-specific Monte Carlo CT dosimetry software ImpactMC, the computational phantom-based dosimetry tool NCICT could provide organ dose estimates within ± 22% for whole-body CT scans acquired with TCM. If better accuracies are required, patient-specific Monte Carlo simulations are recommended. Depending on the organ of interest and the specific CT scanner, SSDE may be a good first estimate of the organ dose.

**Supplementary Information:**

The online version contains supplementary material available at 10.1186/s40658-025-00811-x.

## Introduction

Since its clinical introduction in the 1970s, the use of X-ray computed tomography (CT) has been expanding rapidly [1]. This rising trend in annual number of acquired CT examinations is not confined to the European countries. In the United States an increase of about 20% over the decade 2006–2016 was reported by the NCRP [2]. The observed increase is related to the development of new techniques, protocols and technologies, which makes CT also suitable for screening of lung and colon cancer, and to guide minimally invasive interventional procedures. In addition, its application has extended towards medical imaging domains beyond diagnostic radiology. Hybrid imaging modalities such as PET/CT and SPECT/CT are well-established tools in modern nuclear medicine practice. Depending on the clinical task and the corresponding image quality requirements, a CT scan can be performed for attenuation correction only, anatomical localisation and/or diagnostic purposes. Most hybrid imaging systems employ a CT scanner with full diagnostic capacities and have separate protocols for each clinical purpose. Although the radiation dose from the CT scan may differ and the risks of multi-modality imaging are generally far outweighed by the benefits, it still results in an increased radiation exposure to the patient due to the combined dose from the CT component and the radiopharmaceutical. Together with the growing concern about the long-term effects of radiation exposure, especially the risk of cancer in the paediatric and a large part of the adult population, the need for accurate patient CT dose estimates has increased [3, 4].

The standard dose metrics volume CT dose index (CTDI_vol_) and dose-length product (DLP) are determined as standardised to a 16 or 32 cm diameter IEC dosimetry phantom [5]. Although they are sensitive to changes in CT scan parameters, displayed on the console of the CT scanner and are included in the patient’s dose report, they do not represent the dose to the patient’s organs [6]. They only enable the comparison of different CT protocols and scanners. To estimate patient doses more accurately, the AAPM Task Group introduced the concept of size-specific dose estimate (SSDE) [7, 8]. It incorporates the size of the patient by scaling the CTDI_vol_ with the effective or water equivalent diameter of the patient. Nevertheless, to assess potential radiation risks associated with CT exposure, more accurate individual organ dose estimates are needed.

Different methodologies exist to calculate organ doses. Although the use of physical anthropomorphic phantoms with thermoluminescent dosimeters (TLDs) is a reliable method, it is a very labour-intensive and time-consuming method [9, 10]. Therefore, computational methods were developed as an alternative approach. In general, they make use of pre-calculated tables of organ dose distributions, typically obtained through Monte Carlo simulations, in a given phantom [11]. Some of these easy-to-use dosimetry tools such as CT-Expo [12] make use of mathematical phantoms, which limits their ability to describe detailed human anatomy. Besides, they are mostly also limited in the number of available phantoms. Preferably, dosimetry tools using voxel or hybrid voxel models, which are created from cross-sectional patient CT images resulting in a more realistic representation of the human anatomy, are used. Some examples are WAZA-ARI [13, 14], VirtualDose [15] and NCICT [16, 17]. While the first tool is limited to four adult and five paediatric male and female phantoms, the latter two incorporate a more extensive library of male and female phantoms with various heights and weights. In addition, NCICT also contains a similar library of paediatric phantoms. Although these tools allow an easy and quick organ dose estimation, they are not patient specific. Even a large library of computational hybrid voxel models cannot account for all variations in organ sizes and positions. Therefore, dedicated Monte Carlo (MC) frameworks, such as ImpactMC [18–21], were established that not only allow an accurate description of the X-ray modality but also the implementation of patient-specific voxel models created based on clinically available patient CT data.

Several studies have evaluated the impact of advances in CT dosimetry software on estimates of CT radiation dose [11, 22–25]. However, they are mostly restricted to a comparison with TLD measurements in anthropomorphic phantoms or a comparison of different dosimetry tools using computational phantoms and a limited number of patients, phantoms or CT scanners. Only the study of Papadakis et al. [25] compared CT organ doses assessed through a computational phantom-based and a patient-specific based dosimetry tool with reference to TLD measurements in an anthropomorphic male and female phantom. Nevertheless, their study was restricted to thorax CT examinations on one CT scanner. Therefore, the aim of this study was to compare CT organ doses estimated with a patient-specific Monte Carlo dosimetry tool, using clinical patient CT images and CT data, and estimated with a computational phantom-based dosimetry tool, including phantoms with a range of heights and weights, for diagnostic whole-body CT scans as part of a PET/CT examination on two different standard PET/CT systems with diagnostic CT capacities. This was done for a population of 100 adult patients and phantom models were selected based on patient characteristics such as length and weight. Moreover, special attention was paid to the implementation of tube current modulation in the used phantom-based dosimetry tool, which often means making some assumptions. In addition, the correlation of estimated CT organ doses with patient characteristics and dose indicators was investigated.

## Methods

### Patient selection

Whole-body CT images of one hundred adult patients were collected retrospectively from the institutional Picture Archiving and Communication System (PACS) with the approval of the ethical committee. The reconstructed Field of View (FOV) of the CT scans included the entire cross-section of the patient. In this way, they are suitable for accurate dose estimations. To comply with the current General Data Protection Regulation (GDPR) rules, all CT data was anonymised according to the hospital’s anonymisation policy. Only data concerning patient sex, age, length and weight was kept.

Of all hundred patients, fifty patients underwent a whole-body PET/CT examination on a Siemens Biograph mCT Flow (Siemens Healthineers, Germany) while the other fifty patients underwent a whole-body PET/CT examination on a GE Discovery MI (GE HealthCare, United States). For each PET/CT system, an equal number of male and female patients was selected. They were chosen in such a way as to assure a wide variety in Body Mass Index (BMI) (Table 1).

**Table 1:** Summary of mean (minimum–maximum) age, length, weight and BMI of the study population

Study population	Age(years)	Length(m)	Weight(kg)	BMI (kg/m^2^)
*Siemens Biograph mCT Flow*				
25 females	57(24–86)	1.62(1.50–1.74)	65(39–94)	25(15–36)
25 males	64(33–84)	1.76(1.60–1.95)	79(51–126)	26(16–35)
*GE Discovery MI*				
25 females	54(22–89)	1.64(1.50–1.78)	69(45–96)	26(18–35)
25 males	64(23–90)	1.76(1.63–1.94)	80(53–114)	26(16–41)

### CT scanners and acquisition protocols

All CT scans, either performed on the 40-slice Siemens Biograph mCT Flow PET/CT (Siemens Healthineers, Germany) or the 64-slice GE Discovery MI PET/CT (GE Healthcare, USA), were acquired from head to mid-thigh. Although in nuclear medicine whole-body PET/CT examinations can be performed with a diagnostic or a lower dose localisation CT scan, this study focussed on the estimation of organ doses for diagnostic CT scans. This type of CT scan is, typically, acquired after contrast agent administration. The CT acquisition parameters were retrieved automatically from the DICOM header of the patient CT images with an in-house developed ImageJ/Fiji macro. However, they could also be collected from the protocol on the console of the CT scanner. For both PET/CT systems, the diagnostic CT scans were performed at 120 kV with a rotation time of 0.5 s. While the Siemens CT protocol applied a beam collimation of 19.2 mm and a pitch of 0.7, this was, respectively, 40 mm and 0.984 for the protocol at the GE CT. An overview of the CT scan parameters is given in Table 2.

**Table 2:** Summary of exposure parameters for a diagnostic whole-body CT with tube current modulation at a Siemens Biograph mCT Flow and GE Discovery MI PET/CT

Parameter	Siemens whole-body CT	GE whole-body CT
Tube voltage (kV)	120	120
Tube current (mA)	ATCM*	ATCM*
Rotation time (s)	0.5	0.5
Pitch	0.7	0.984
Beam collimation (mm)	19.2	40.0
Scan FOV (mm)	500	500
Scan start	head	head
Scan end	mid-thigh	mid-thigh

^*^ Automatic tube current modulation

Both PET/CT systems apply tube current modulation (TCM) for whole-body CT scans. The TCM system available on the Siemens CT scanner is *CARE Dose4D*. This means that the tube current value in the DICOM header of each reconstructed slice is the average of the applied angularly and longitudinally modulated values [26–31]. The tube current is modulated based on the patient’s 2D projection radiograph, i.e. topogram, and the applied quality reference mAs that defines the desired image quality on a standard-sized patient. GE, on the other hand, uses *Auto mA* and *Smart mA*. While Auto mA enables tube current modulation in the longitudinal z-direction, Smart mA enables angular modulation in the x- and y-direction as well [32, 33]. Based on the patient’s 2D projection radiograph, i.e. scout, and the selected noise index, the system computes the required tube current values to be used.

In addition, the CTDI_vol_ and DLP were retrieved from the Radiation Dose Structured Report (RDSR). This report, which also contains the applied scan parameters for each acquired CT scan, was sent to the PACS together with the reconstructed image series.

### Patient-specific dosimetry

**Fig. 1:**
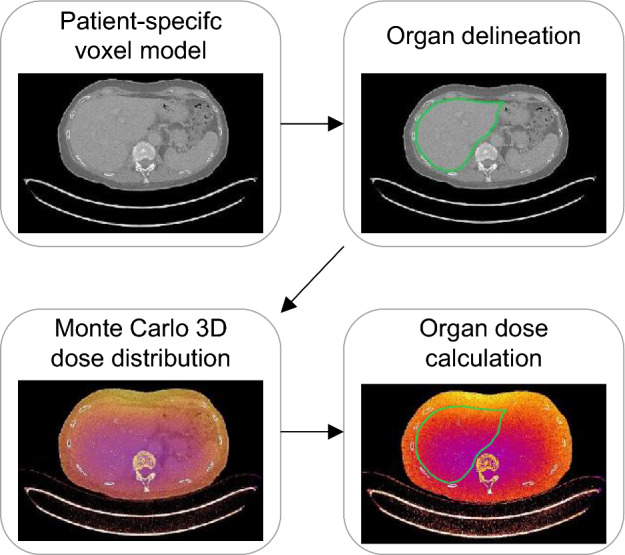
Overview of the workflow to estimate patient-specific organ doses with ImpactMC – Whole-body CT images were used to create patient-specific 3D voxel models. Individual organs were delineated on the original CT images. The organ segmentations were then used to calculate mean organ doses on the output images of the Monte Carlo simulations

To estimate patient-specific CT organ doses, Monte Carlo (MC) simulations were performed with ImpactMC 1.6 (CT Imaging GmbH, Erlangen Germany), which was validated by several research groups using either the IEC CT body and/or head dosimetry phantom or anthropomorphic phantoms [18–21]. It combines Monte Carlo algorithms with scanner specific parameters such as geometric, spectral and shaped filter characteristics, and patient-specific voxel models based on patient CT images. In this way, the software calculates individualised 3D dose distributions, considering all relevant photon interaction processes [19, 21]. The ImpactMC software has proven to estimate CT doses within 10% from physical dose measurements for several homogeneous and heterogeneous phantoms and various CT scanners [19]. Delineation of the organs of interest makes it then possible to estimate patient-specific organ and tissue doses. An overview of the complete workflow is given in Fig. 1.

#### Voxel models

Based on the data of the 512 × 512 DICOM images, a patient-specific 3D whole-body voxel model was created for each of the hundred patients included in this study. The CT images were not resampled. The voxel models reconstructed from the Siemens PET/CT have a voxel size of 0.9727 × 0.9727 × 3 mm^3^ while this is 0.9766 × 0.9766 × 2.5 mm^3^ for the GE PET/CT voxel models.

#### Delineation of organs

The radiosensitive organs and tissues of interest were delineated on the whole-body CT images by a medical physicist. For this purpose, the open source software tools Fiji/ImageJ [34, 35] and 3D Slicer [36] were used. For the lungs and liver, the regions of interest (ROIs) were obtained semi-automatically while for the breast (female patients), heart, kidneys, thyroid and oesophagus manual delineation was performed.

#### Monte carlo dose simulations

To perform scanner specific dose computations, the CT part of a Siemens Biograph mCT Flow and a GE Discovery MI was modelled. Geometrical specifications, such as the focus to isocenter distance (Siemens: 595 mm; GE: 541 mm) and fan angle (Siemens: 0.7955; GE: 0.8658), were derived from specific data elements, DICOM tags, in the DICOM header of the CT images. However, they could also be extracted from the technical reference manuals of the systems. To specify the respective X-ray spectra, the methodology described by Turner et al*.* [37] for equivalent energy spectra in CT was used. Therefore, the first half-value layer at a tube voltage of 120 kV was derived experimentally for each CT scanner. Equivalent spectra were then generated using a MATLAB code (Mathworks, USA) with added SPEKTR tool [38, 39]. The bowtie filter profiles were characterised based on dose measurements. For this, a calibrated pencil beam ionisation chamber (Model 10X6-3CT, Radcal Corporation, USA) was moved in 1 cm intervals from the isocenter while keeping the X-ray tube stationary [37]. In addition, the air kerma free-in-air in the isocenter of the CT was measured to calibrate the Monte Carlo simulation software.

In this study, diagnostic whole-body CT examinations, from head to mid-thigh, were simulated. For each patient, the CT scan parameters of the PET/CT system on which the CT scan was performed were applied (Table 2). To integrate tube current modulation, the tube current value from the DICOM header of each reconstructed image was extracted using an in-house developed Fiji/ImageJ macro. To ensure the speed and accuracy of the Monte Carlo simulation, the number of interacting photons was chosen to be 10^10^ for all simulations. In order to convert the CT values of the input whole-body patient CT images into density values the standard conversion curve incorporated in the ImpactMC software was used [40].

#### Organ dose calculation

A Monte Carlo dose simulation with ImpactMC results in a 3D dose distribution based on the physical properties (i.e. attenuation, composition and size) of the input patient CT scan. Overlaying the contours of each organ on the corresponding slices of the dose distribution results in an estimation of patient-specific organ doses *D*_*T*_ which were determined as follows:

$${D}_{T}=\sum_{i=1}^{N}\left({f}_{i,T}\cdot {M}_{i,T}\right) with {f}_{i,T}=\frac{{A}_{i,T}}{{\sum }_{i=1}^{N}{A}_{i,T}}$$

where *M*_*i,T*_ is the mean dose within the contour at slice *i* of organ *T*, *N* the total number of slices that contain contours of organ *T* and *f*_*i,T*_ the fractional area of each organ contour (with *A*_*i,T*_ the area within the contour at slice *i* of organ *T)*. To enable unsupervised organ dose calculation, an algorithm was implemented in Fiji/ImageJ.

### Computational phantom-based dosimetry

**Fig. 2:**
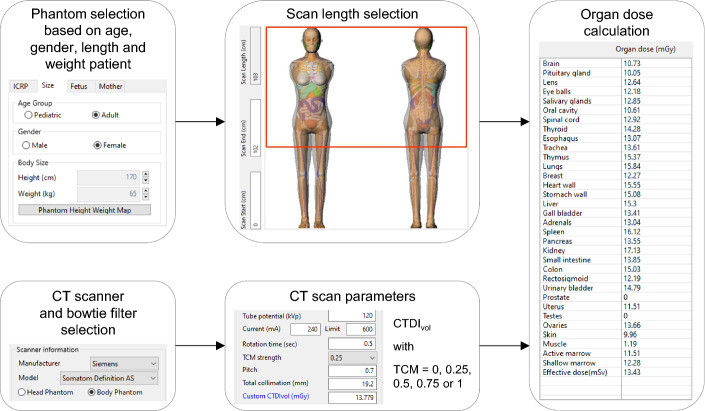
Overview of the workflow to estimate organ doses with NCICT–Based on the age, sex, length and weight of the patient the most suitable phantom was selected. The whole-body CT scan length was chosen similar to the scan length of the original patient CT scan. A CT scanner with similar characteristics as the CT part of the PET/CT system was selected from the same vendor. For whole-body CT imaging the body filter was chosen. The tube voltage, pitch, collimation and CTDI_vol_ of the CT examination were given as CT scan parameters to the software. A TCM strength of 0.25 was applied for scans performed with tube current modulation. Organ doses were then calculated for various organs and tissues

To estimate organ doses, easy-to-use dosimetry tools exist as well. In this study, the computational phantom-based dosimetry tool NCICT 3.0 (National Cancer Institute, Bethesda USA) was used [16, 17]. Besides the ICRP reference paediatric (newborn, one year, five year, ten year and fifteen year old) and adult male and female phantoms, it has a phantom library that includes male and female paediatric and adult phantoms with a range of heights (paediatric: 85–185 cm; adult: 150–190 cm) and weights (paediatric: 10–125 kg; adult: 40–140 kg). In addition, pregnant female phantoms at different gestational ages are available. Together with a pre-calculated dose database, obtained through Monte Carlo simulation of a reference CT scanner combined with the computational human phantom library, NCICT allows the calculation of the radiation dose delivered to 31 organs and tissues [17]. The results in the dose database were validated through experimental measurements by several research groups [24, 41, 42]. An overview of the workflow to estimate organ or tissue doses with NCICT is shown in Fig. 2.

#### Phantom selection

NCICT 3.0 offers a library of 100 male and 93 female adult phantoms with a range of heights (male: 160–190 cm; female: 150–175 cm) and weights (male: 50–140 kg; female: 40–135 kg). Within these ranges the phantom dimensions vary by 5 cm or 5 kg, respectively. Therefore, for each adult patient included in this study, the most suitable phantom was selected manually based on the patient’s length and weight that were embedded in the DICOM header of the CT images.

#### CT scanner selection

Although multiple CT scanners from different vendors, including Siemens and GE, are incorporated in NCICT 3.0 it does not cover the specific CT systems employed by the Siemens Biograph mCT Flow and GE Discovery MI PET/CT. Therefore, from the same vendor, a CT scanner with similar characteristics such as the X-ray tube, bowtie filter, the number of detector rows, collimation width, TCM system and tube voltage range was selected. For the patients that underwent a whole-body examination on the Siemens and GE PET/CT, respectively, the *Siemens Somatom Definition AS* and *GE Discovery CT HD 750* CT scanner model were used to estimate organ and tissue doses with NCICT. To assess whether this choice influences the accuracy of the dose estimates, dose calculations using a second available CT model were performed as well. According to the same CT characteristics, the *Siemens Somatom Sensation 40* and *GE Optima 600* were chosen for this.

#### Organ dose calculation

To calculate whole-body CT organ doses, the appropriate bowtie filter needs to be selected. In this case, this corresponds to the ‘Body Phantom’ filter (Fig. 2). The applied CT scan length was identical to the clinical scan length, which was determined based on the information embedded in the DICOM images. The CT scan parameters were those of the clinical whole-body CT protocols as given in Table 2. However, because the CTDI_vol_ of the examination was available, this value could be entered directly into the user interface. It overrules all other scan parameters except the tube voltage and tube current modulation (TCM) strength. The latter is based on predetermined TCM profiles and can have a value between zero and unity [17]. Organ doses were retrieved for the lungs, liver, breast (female patients), heart, kidneys, thyroid and oesophagus. For each performed CT acquisition, the real clinical CTDI_vol_ was used and a TCM strength of 0.25 was applied. To study the implementation of the TCM system, organ doses were also estimated using a TCM strength of 0, 0.5, 0.75 and 1.

To obtain organ and tissue doses for a large-scale patient group, NCICT offers the possibility to import the list of required parameters and automatically export the calculated organ doses by a batch routine. This speeds up the organ dose calculation.

### Data analysis

For each patient, PET/CT system and dosimetry tool, absolute organ doses and organ doses normalised to CTDI_vol_ of a diagnostic whole-body CT with TCM were determined as described before.

#### Comparison of organ dose estimations

To enable a comparison of organ doses between patient-specific and computational phantom-based dosimetry, the patients were classified based on their BMI: underweight (BMI < 18.5 kg/m^2^), healthy weight (BMI between 18.5 and 24.9 kg/m^2^), overweight (BMI between 25.0 and 29.9 kg/m^2^) and obesity (BMI ≥ 30.0 kg/m^2^). In total, ten patients were categorised in the underweight group while 40, 28 and 22 patients belonged to the healthy weight, overweight and obesity group, respectively.

For each PET/CT system, organ of interest and BMI category, the differences in organ doses estimated with ImpactMC and NCICT were calculated. In this way, the influence of the applied TCM strength in NCICT on the accuracy of simulated organ doses between patient-specific and computational phantom-based dosimetry tools was studied. Besides, it allows to explore the overall accuracy of organ doses estimated using computational phantoms instead of patient-specific voxel models.

Two-way mixed model intraclass correlation coefficients (ICC) for single measures and absolute agreement, and their 95% confidence intervals, were calculated to quantify the agreement between both dosimetry tools. This was done for each PET/CT system and organ of interest with and without differentiating in BMI categories. In addition, bivariate least square (also known as Deming regression) regression coefficients were calculated using a measurement error of 10% and 25% for ImpactMC and NCICT, respectively [19]. To increase the number of datapoints, no distinction in BMI categories was made for the Deming regression analysis. All statistical calculations were performed with R, a free software environment for statistical computing and graphics [43].

#### Correlation with patient characteristics and dose indicators

To assess whether organ dose differences are related to variations in patient length, weight or BMI, regression analysis was performed and the coefficient of determination, R^2^, was used as a measure to assess the strength of the correlation. No distinction was made between different BMI categories.

Finally, the obtained organ doses were correlated with the size-specific dose estimate (SSDE), which considers the X-ray attenuation of the patient. According to the AAPM Report No. 220 [8], the size-specific dose estimate of each patient included in this study was obtained by multiplying the CTDI_vol_ of the CT examination with the water equivalent diameter, D_W_, of the patient. The latter is calculated as:

$${D}_{W}(z)=2\cdot \sqrt{\left[\frac{1}{100}\overline{{CT(x,y)}_{ROI}}+1\right]\cdot \frac{{A}_{ROI}}{\pi }}$$

where $\overline{{CT(x,y)}_{ROI}}$ is the average Hounsfield Unit (HU) value over each $x,y$ location in the ROI that contains the imaged patient in one slice and ${A}_{ROI}$ the area of the ROI. The SSDE at that slice, SSDE (z), is then calculated as:

$$SSDE(z)=a\cdot {e}^{-b\cdot {D}_{w}}\cdot {CTDI}_{vol}$$

where a and b are exponential fit coefficients, depending on the diameter of the PMMA phantom (either 16 or 32 cm) used to measure the CTDI_vol_ [8]. The mean water equivalent diameter and SSDE over the entire scan range is then determined as:

$$\overline{{D}_{W}}=\frac{\sum_{z=1}^{N}{D}_{W}}{N}$$

$$\overline{SSDE}=\frac{\sum_{z=1}^{N}SSDE(z)}{N}$$

where *N* is the total number of images in the scan range. Due to the large number of patients, an ImageJ algorithm was developed to automate these calculations using the patient’s body contours previously delineated on the CT images with the help of 3D Slicer and a developed ImageJ macro.

Correlations with SSDE and coefficients of determination were determined through regression analysis for organ doses obtained with ImpactMC as well as with NCICT. Note that in both cases the same size-specific dose estimates, related to the real patients, were used. This means that we did not use the SSDE of the computational phantom model when correlating them with the organ doses from NCICT.

## Results

Organ doses, of patients undergoing a whole-body CT scan at 120 kV with tube current modulation, were calculated for the breast, heart, liver, lungs, kidneys, thyroid and oesophagus using the patient-specific Monte Carlo dosimetry software ImpactMC and the phantom-based dosimetry tool NCICT 3.0. In addition, the organ doses were normalised to the CTDI_vol_ of the respective CT examination.

### Influence of CT scanner model on organ dose estimates in NCICT

No differences were observed between organ doses obtained with NCICT when the Siemens Somatom Sensation 40 CT scanner was selected instead of the Siemens Somatom Definition AS. Identical organ dose estimations were also found for the GE Discovery CT HD 750 and GE Optima 600. This was seen for each patient and each selected TCM strength.

### Accuracy of organ dose estimations

For each patient, the percentage difference in CT organ doses between estimations obtained with ImpactMC and NCICT was calculated. This was done for each organ of interest and for the diagnostic whole-body CT examinations performed with tube current modulation on the Siemens and GE PET/CT separately.

#### Influence of TCM strength

**Fig. 3:**
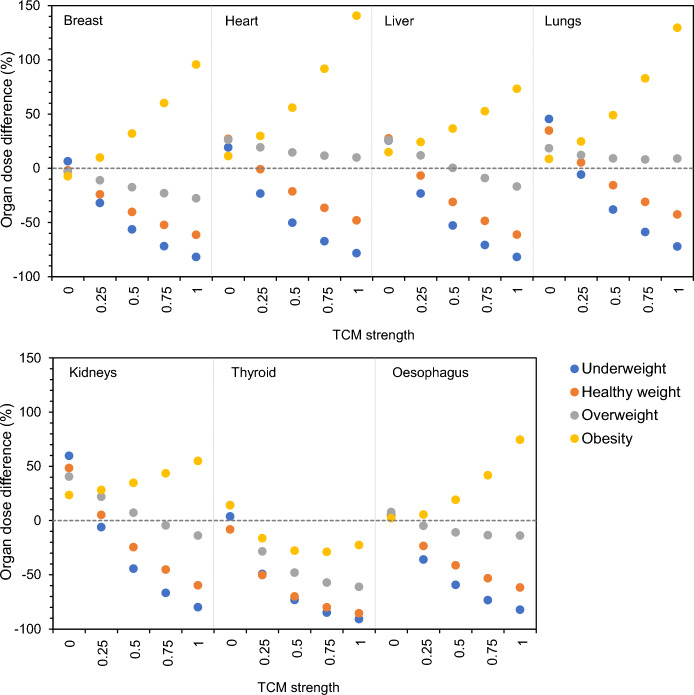
Percentage difference in mean organ dose, for each BMI category, of a whole-body CT scan at 120 kV on a Siemens Biograph mCT Flow PET/CT simulated with tube current modulation (TCM) using ImpactMC and simulated with different TCM strengths (0, 0.25, 0.5, 0.75 and 1) using NCICT 3.0

Using NCICT, tube current modulation can be implemented based on predefined tube current modulation (TCM) strength profiles. To define which applied TCM strength value (0, 0.25, 0.5, 0.75 or 1) results in organ dose estimations that differ minimally from those obtained with patient-specific Monte Carlo tools as ImpactMC and thus mimics the clinical tube current modulation the best, organ doses were established for each possibility. For each situation, the percentage differences with CT organ doses estimated with ImpactMC were determined. Figure 3 shows the mean results for each organ and BMI category of patients undergoing a whole-body CT acquisition on a Siemens Biograph mCT Flow PET/CT. Overall, an increase in mean CT organ dose difference is seen with increasing TCM strength. Although for overweight patients, the dose results for the heart, liver, lungs and kidneys suggest that a TCM strength of 0.5 should be used, the results of the other organs and BMI categories suggest the application of a TCM strength of zero or 0.25. Similar observations were found for the whole-body CT examination on a GE Discovery MI PET/CT (Figure S1, Additional File 1).

**Fig. 4:**
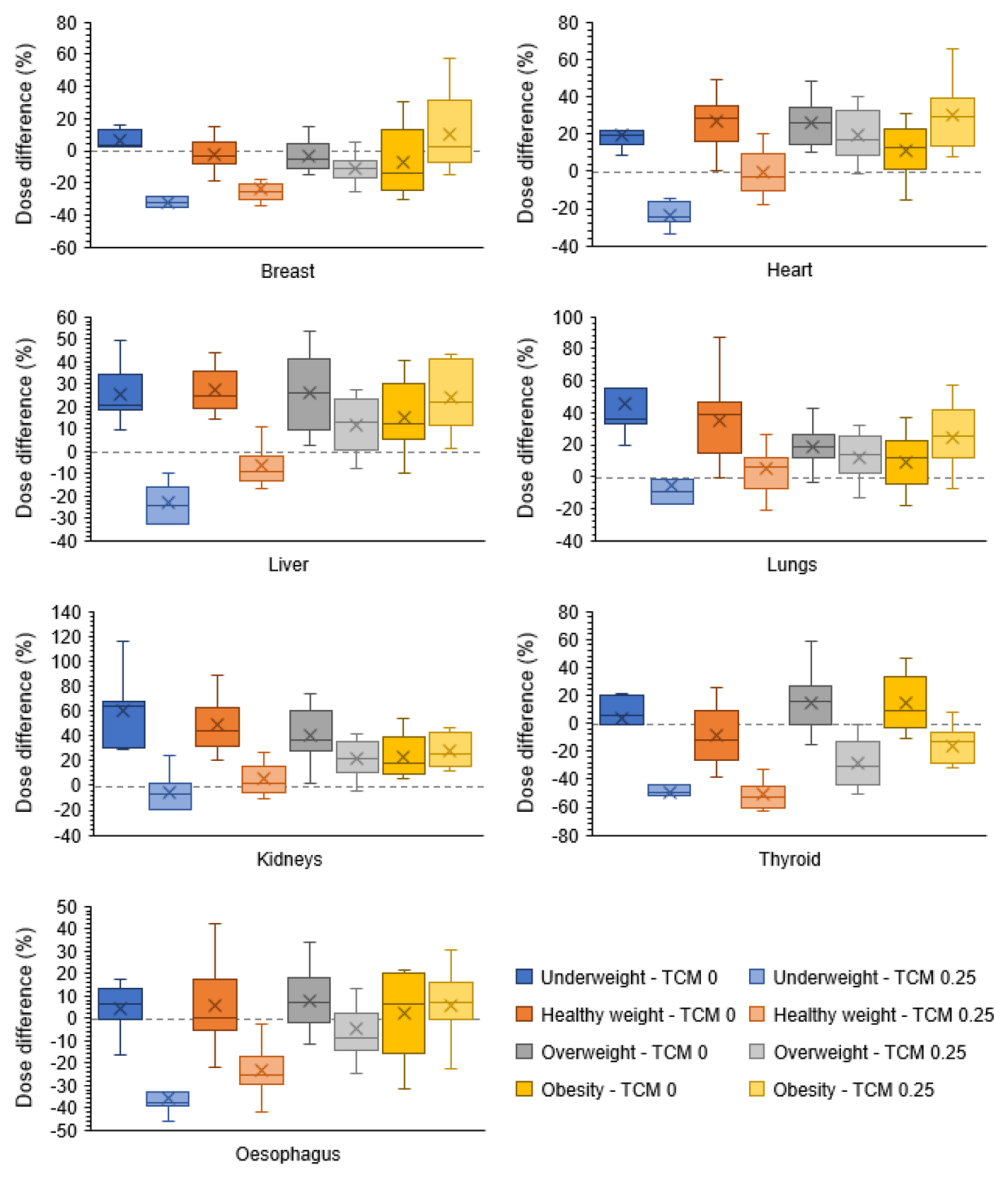
Distribution of the percentage difference in organ dose, for each BMI category, of a whole-body CT scan on a Siemens Biograph mCT Flow PET/CT simulated with NCICT applying a tube current modulation strength of ‘0’ or ‘0.25’ and simulated with ImpactMC using the clinically applied tube current modulation values

Because a TCM strength of zero corresponds with a CT acquisition without tube current modulation, the distribution of the percentage dose differences for each organ and BMI category was investigated as well. Figure 4 visualises these distributions for the organ dose comparisons where a TCM strength of zero or 0.25 was used in NCICT. As can be seen, the difference between the minimum and maximum observed dose difference is smaller when in NCICT a TCM strength of 0.25 is applied to estimate the CT organ doses. For the other PET/CT system in this study, a GE Discovery MI, similar trends were seen (Figure S2, Additional File 1).

Based on these observations, a TCM strength of 0.25 was used when estimating CT organ doses of examinations applying tube current modulation with NCICT.

#### Mean organ dose and accuracy range

Table 3 lists the mean organ doses normalised to CTDI_vol_ and their corresponding standard deviation assessed through ImpactMC and NCICT, for whole-body CT examinations performed with tube current modulation on a Siemens Biograph mCT Flow and GE Discovery MI PET/CT, for the patients classified in the underweight and healthy weight category. A TCM strength of 0.25 was applied when using NCICT. In addition, the mean, minimum and maximum percentage dose differences between organ doses estimated with ImpactMC and NCICT are given. Negative percentage dose differences indicate an underestimation of the organ dose by NCICT. This means that the organ dose estimated by NCICT is smaller than the one estimated by ImpactMC. The results for the patients classified in the overweight and obese category are given in Table 4.

**Table 3:** Underweight (BMI < 18.5 kg/m^2^) and healthy weight (BMI between 18.5 and 24.9 kg/m^2^) patients – Mean organ dose normalised to CTDI_vol_ (mGy/mGy) and standard deviation obtained with ImpactMC and NCICT for whole-body CT scans applying tube current modulation (TCM), and percentage organ dose difference (mean, minimum and maximum) between ImpactMC and NCICT

	Organ dose normalised to CTDI_vol_ (mGy/mGy)				Organ dose difference (%)	
	Underweight		Healthy weight		Underweight	Healthy weight
	ImpactMC	NCICT	ImpactMC	NCICT		
*Siemens Biograph mCT Flow*						
Breast	1.27 ± 0.07	0.88 ± 0.05	1.26 ± 0.14	0.95 ± 0.07	− 32.0(− 35.4; − 28.7)	− 24.1(− 34.5; − 4.6)
Heart	1.42 ± 0.13	1.08 ± 0.05	1.18 ± 0.12	1.16 ± 0.06	− 23.4(− 33.8; − 14.9)	− 0.9(− 18.0; 19.8)
Liver	1.40 ± 0.17	1.06 ± 0.02	1.20 ± 0.10	1.11 ± 0.02	− 23.2(− 32.3; − 9.6)	− 6.7(− 16.8; 10.5)
Lungs	1.18 ± 0.15	1.09 ± 0.05	1.13 ± 0.17	1.16 ± 0.05	− 5.9(− 17.5; 22.5)	5.3(− 21.3; 45.1)
Kidneys	1.26 ± 0.16	1.16 ± 0.03	1.15 ± 0.12	1.20 ± 0.03	− 6.1(− 18.8; 23.6)	5.2(− 10.6; 26.5)
Thyroid	2.32 ± 0.58	1.12 ± 0.11	2.52 ± 0.53	1.20 ± 0.14	− 49.2(− 72.3;− 28.9)	− 50.3(− 63.2; − 18.7)
Oesophagus	1.49 ± 0.12	0.95 ± 0.05	1.30 ± 0.18	0.98 ± 0.05	− 36.0(− 46.3; − 20.6)	− 23.4(− 41.9; 9.0)
*GE Discovery MI*						
Breast	1.53 ± 0.00	0.87 ± 0.00	1.43 ± 0.15	0.94 ± 0.07	− 43.0(− 43.0; − 43.0)	− 33.8( − 44.2; − 14.5)
Heart	1.63 ± 0.21	1.08 ± 0.02	1.51 ± 0.19	1.13 ± 0.06	− 32.6(− 40.1; − 22.6)	− 24.0(− 36.0; 0.3)
Liver	1.54 ± 0.25	1.08 ± 0.02	1.43 ± 0.19	1.10 ± 0.02	− 28.5(− 40.0; − 19.3)	− 21.9(− 40.0; − 1.7)
Lungs	1.38 ± 0.16	1.08 ± 0.00	1.48 ± 0.14	1.14 ± 0.05	− 21.0(− 30.8; − 13.3)	− 22.1(− 41.7; 1.6)
Kidneys	1.79 ± 0.26	1.17 ± 0.04	1.46 ± 0.30	1.20 ± 0.04	− 33.5(− 42.2;− 22.7)	− 15.4(− 52.9; 11.7)
Thyroid	3.03 ± 0.65	1.09 ± 0.06	2.48 ± 0.60	1.24 ± 0.13	− 63.0(− 69.9;− 53.9)	− 47.3(− 72.5;-11.6)
Oesophagus	1.69 ± 0.08	0.93 ± 0.00	1.49 ± 0.22	0.97 ± 0.05	− 45.0(− 47.8;− 42.7)	− 33.1(− 55.7; − 4.4)

**Table 4:** Overweight (BMI between 25 and 29.9 kg/m^2^) and obese (BMI ≥ 30 kg/m^2^) patients – Mean organ dose normalised to CTDI_vol_ (mGy/mGy) and standard deviation obtained with ImpactMC and NCICT for whole-body CT scans applying tube current modulation (TCM), and percentage organ dose difference (mean, minimum and maximum) between ImpactMC and NCICT

	Organ dose normalised to CTDI_vol_ (mGy/mGy)				Organ dose difference (%)	
	Overweight		Obese		Overweight	Obese
	ImpactMC	NCICT	ImpactMC	NCICT		
*Siemens Biograph mCT Flow*						
Breast	1.19 ± 0.19	1.04 ± 0.10	1.25 ± 0.27	1.32 ± 0.09	− 11.1(− 25.6; 5.5)	9.8(− 15.3; 57.9)
Heart	1.00 ± 0.11	1.19 ± 0.08	1.00 ± 0.09	1.28 ± 0.09	19.3(− 1.5; 39.9)	29.7(7.9; 65.6)
Liver	1.03 ± 0.12	1.14 ± 0.03	0.97 ± 0.10	1.19 ± 0.05	− 6.7(− 16.8; 10.5)	24.2(0.8; 43.2)
Lungs	1.09 ± 0.14	1.21 ± 0.06	1.05 ± 0.17	1.28 ± 0.07	12.2(− 13.7; 31.7)	24.7(− 7.2;56.9)
Kidneys	1.01 ± 0.11	1.22 ± 0.03	0.96 ± 0.09	1.22 ± 0.02	22.0(− 4.1; 41.5)	28.2(11.5;46.9)
Thyroid	1.90 ± 0.34	1.32 ± 0.18	1.68 ± 0.28	1.38 ± 0.15	− 28.4(− 50.7; − 0.8)	− 16.3(− 31.4; 8.1)
Oesophagus	1.06 ± 0.14	0.99 ± 0.05	1.00 ± 0.21	1.03 ± 0.06	− 4.9(− 24.6; 32.6)	5.5(− 31.2; 30.3)
*GE Discovery MI*						
Breast	1.52 ± 0.25	1.04 ± 0.08	1.44 ± 0.14	1.18 ± 0.13	− 30.4(− 46.0; − 7.4)	− 17.9(− 28.1; 3.7)
Heart	1.33 ± 0.16	1.22 ± 0.07	1.23 ± 0.11	1.24 ± 0.08	− 7.3(− 19.2; 22.6)	1.7(− 14.6; 25.5)
Liver	1.33 ± 0.20	1.15 ± 0.03	1.24 ± 0.13	1.16 ± 0.03	− 11.5(− 30.6; 22.1)	-4.9(− 22.1; 18.8)
Lungs	1.35 ± 0.12	1.24 ± 0.05	1.30 ± 0.09	1.27 ± 0.06	− 7.5(− 23.7; 15.3)	− 1.7(− 12.4; 21.0)
Kidneys	1.34 ± 0.21	1.23 ± 0.03	1.22 ± 0.13	1.23 ± 0.03	− 6.3(− 37.5; 23.9)	2.1(− 12.8; 21.7)
Thyroid	1.86 ± 0.25	1.36 ± 0.18	1.52 ± 0.14	1.38 ± 0.21	− 26.3(− 42.6; − 4.7)	− 7.7(− 38.8; 20.5)
Oesophagus	1.25 ± 0.10	1.02 ± 0.05	1.11 ± 0.07	1.02 ± 0.06	− 17.7(− 31.5; − 5.9)	− 8.4(− 17.6; 12.2)

In general, smaller standard deviations were found for organ doses estimated with NCICT. However, some exceptions are observed such as for the thyroid dose for the whole-body CT examination on the GE Discovery MI PET/CT.

#### Correlation with patient length, weight and BMI

Through linear regression analysis the relationship between the observed percentage organ dose differences and patient characteristics such as length, weight and BMI was investigated. No correlations were observed with patient length (R^2^ < 0.1) while very weak to moderate correlations with patient weight (0.2 < R^2^ < 0.6) and BMI (0.2 < R^2^ < 0.7) were found. However, the observed moderate correlations were in the minority. This was seen for both PET/CT systems.

### Organ dose correlations

For each PET/CT system and organ of interest, the organ doses estimated with ImpactMC and NCICT were correlated with each other and with the size-specific dose estimate (SSDE).

#### Relation between organ doses from ImpactMC and NCICT

When making a distinction in BMI category, intraclass correlations coefficients (ICC) and their 95% confidence intervals vary considerably (Additional File 1, Table S1 and Table S2). Generally, ICC indicate moderate or excellent agreement between ImpactMC and NCICT for the breast, heart, liver, lung and kidney dose of healthy weight and overweight patients undergoing a whole-body CT on both the Siemens and GE PET/CT system [44]. In addition, similar results were found for the oesophagus and thyroid on the GE PET/CT. Nevertheless, the 95% confidence interval rather indicate a level of reliability ranging from poor or moderate to good and excellent. For underweight and obese patients, the level of reliability ranges from poor to moderate, good or excellent depending on the organ of interest. This is observed for both PET/CT systems.

Low intraclass correlation coefficients can be related to small numbers of patients included in certain BMI categories and thus wrongly result in a low degree of measurement agreement [44]. Therefore, ICC were also calculated disregarding differences in patient BMI (Additional File 1, Table S3). For the GE PET/CT, a good to excellent agreement was found between the doses estimated with ImpactMC and NCICT for all organs of interest except the thyroid. For the latter, a moderate to good level of reliability was found. Overall, lower ICC were found for the organ doses related to the CT acquisition of the Siemens Biograph mCT Flow PET/CT. While moderate to good levels of agreement were observed for the breast, heart, liver, lung and kidney doses, poor to moderate levels were found for the oesophagus and thyroid.

**Fig. 5:**
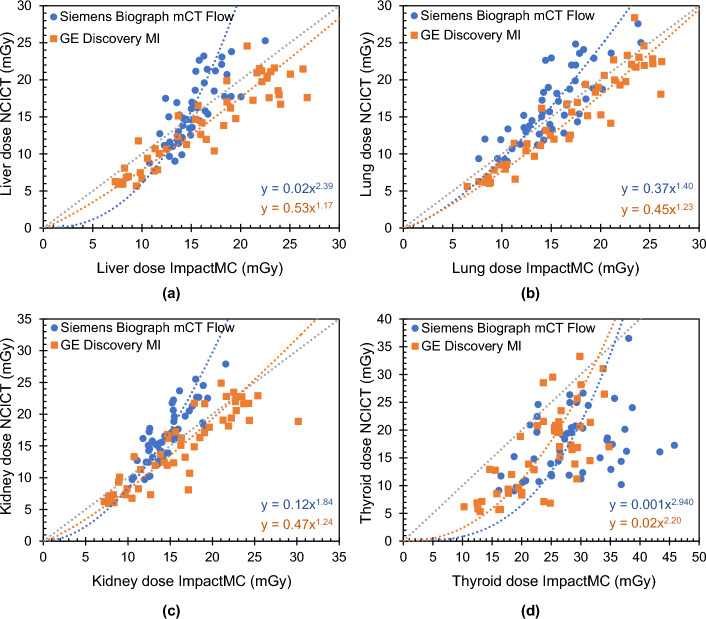
Relationship between the estimated organ dose calculated with the patient-specific Monte Carlo dosimetry tool ImpactMC and the phantom-based dosimetry software NCICT for a whole-body CT examination on a Siemens Biograph mCT Flow and GE Discovery MI PET/CT acquired with tube current modulation for **a** the liver, **b** the lungs, **c** the kidneys and **d** the thyroid. The added grey dashed line represents the curve that would be followed when both dose calculation methods give the same result

Through bivariate least square or Deming regression analysis the relation between the organ doses estimated with the patient-specific Monte Carlo approach ImpactMC and the phantom-based dosimetry tool NCICT was investigated. For all organs and both PET/CT system a relation of the following form was found

$$y=a\cdot {x}^{b}$$

where the variables *x* and *y* correspond to the organ dose estimated with ImpactMC and NCICT, respectively. Both variables are expressed in mGy. For each regression curve, the regression coefficients *a* and *b* were estimated. Figure 5 displays the Denim regression curves for the liver, lung, kidney and thyroid dose for whole-body CT scans acquired with tube current modulation on both PET/CT systems. For the breast, heart and oesophagus the results are shown in Figure S3 (Additional File 1).

#### Correlation of organ doses with SSDE

As described before, the size-specific dose estimate considers the size of the patient and the CT dose parameter CTDI_vol_. The correlation between organ dose and SSDE was determined through regression analysis. Hereby, the assumption was made that no CT scan results in a CTDI_vol_ and organ dose value of zero. The regression curve should thus go through the origin. The estimated regression function was of the linear form

$$y=m\bullet x$$

where the independent variable, *x*, corresponds to the size-specific dose estimate (SSDE) expressed in mGy and the dependent variable, *y*, corresponds to the organ dose, also expressed in mGy, estimated either with ImpactMC or with NCICT. For each linear regression curve, the parameter *m* was estimated.

**Fig. 6:**
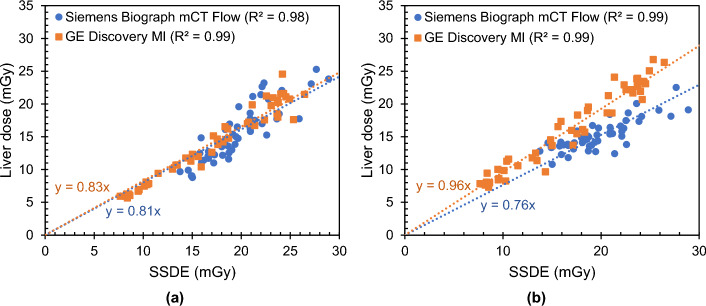
Estimated liver dose as a function of the size-specific dose estimate (SSDE) for a CT scan at 120 kV with tube current modulation as part of a whole-body PET/CT examination on a Siemens Biograph mCT Flow and GE Discovery MI PET/CT. **a** Organ dose estimation with the phantom-based CT dosimetry tool NCICT and **b** organ dose estimation with the patient-specific CT dosimetry tool ImpactMC

**Fig. 7:**
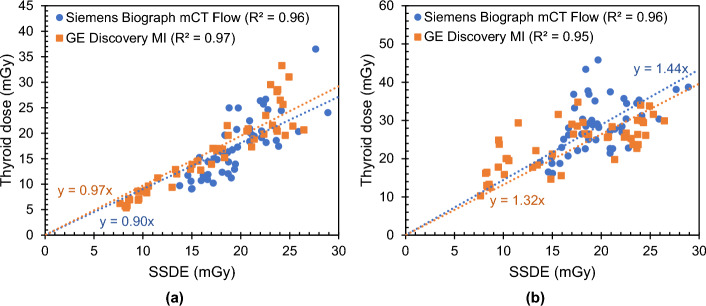
Estimated thyroid dose as a function of the size-specific dose estimate (SSDE) for a CT scan at 120 kV with tube current modulation as part of a whole-body PET/CT examination on a Siemens Biograph mCT Flow and GE Discovery MI PET/CT. **a** Organ dose estimation with the phantom-based CT dosimetry tool NCICT and **b** organ dose estimation with the patient-specific CT dosimetry tool ImpactMC

Figures 6 and 7 show the estimated organ doses as a function of the size-specific dose estimate for, respectively, the liver and thyroid of a whole-body CT scan with tube current modulation simulated with ImpactMC and NCICT. For the breast, heart, lungs, kidneys and oesophagus the results are shown in Figure S4–S8 (Additional File 1). A very strong correlation (0.93 < R^2^ < 1.00) was found for each organ, irrespective of the CT scanner.

## Discussion

Monte Carlo frameworks, such as ImpactMC, are the gold standard to perform patient-specific CT dosimetry. They not only allow an accurate description of the CT scanner, but also the implementation of anatomical 3D models that are based on the patient’s clinical CT images. To implement patient-specific Monte Carlo dose simulations within the clinic, first the CT scanner needs to be characterised. Some characteristics such as the focus to isocenter distance and fan angle can be retrieved from the technical reference manual of the system. To model the X-ray spectrum and bowtie filter, the necessary manufacturer’s data is often unavailable. However, half-value layer and dose measurements, which can be performed without special equipment, provide sufficient input to obtain equivalent X-ray spectra and bowtie filter profiles, respectively. As described in a previous study, Monte Carlo simulations with ImpactMC then result in estimated CT organ doses that deviate less than 3% from those obtained using the more accurate data from the manufacturer [45]. To model the X-ray spectrum, a spectrum generator such as SpekCalc [46] can be used as well. Although this results in a small loss of accuracy, observed CT organ dose differences were still within 6% [45]. Secondly, the CT scan parameters such as rotation time, pitch and applied beam collimation, which can be extracted from the CT protocol on the CT scanner or from the DICOM header of the patient’s CT images, need to be given. A major advantage of patient-specific Monte Carlo simulation tools is the integration of the patient-specific tube current modulation (TCM) profile based on the tube current values embedded in the DICOM header of each reconstructed image. While the time needed to run one Monte Carlo simulation increases with the number of simulated photons and the scan length of the CT examination, the speed of the Monte Carlo dose simulation is primarily determined by the computing capacity of the computer on which it is performed. Finally, organ segmentation may be very time consuming. However, the development of automatic segmentation tools, such as TotalSegmentator [47], and advances in deep learning, create a lot of opportunities to speed up this process.

Computational phantom-based dosimetry tools using voxel or hybrid voxel models, on the other hand, allow an easy and quick estimation of organ doses. Among the available software applications, CT dosimetry tools exist with a phantom library beyond the standard ICRP phantoms. NCICT 3.0, used in this study, is such a dosimetry tool. As described, it contains a library of 100 male and 93 female adult phantoms with a range of heights and weights [17]. In addition, it has a similarly sized library of paediatric phantoms. As observed, the choice of CT scanner does not influence the calculated organ dose estimations when the CTDI_vol_ of the CT examination was entered directly into the user interface. This was seen for both vendors and all patient CT scans included in this study. When looking at the regression curves with the size-specific dose estimate (SSDE) then something else even stands out. For each organ separately, the slopes of the regression curves are nearly identical for both CT devices. It indicates that, besides the CT scan range, the only decisive exposure parameters are the applied tube voltage, TCM strength and CTDI_vol_ [11]. Thus, when the CTDI_vol_ is entered it does not matter which type of CT scanner, or which manufacturer, is selected in the NCICT software. Previous studies, such as the one of Giansante et al. [24], already recognised that in this case the NCICT code is entirely based on a reference CT scanner and relies on the fact that CTDI_vol_-normalised organ doses do not depend on the CT scanner [17]. The small deviations observed in the slope values of the regression curves are probably related to the different patient population, and thus different phantom-based population, which was used for both PET/CT systems. On the other hand, also the pitch and beam collimation of the whole-body CT protocols differ, which influences the CTDI_vol_ values. Moreover, the applied TCM system may play a role as well.

The main goal of this study was to compare the organ doses estimated with the patient-specific Monte Carlo software ImpactMC to the doses obtained with the phantom-based dosimetry tool NCICT. While ImpactMC offers the opportunity to implement tube current modulation based on the tube current values embedded in the DICOM header of the CT images, thus incorporating the realistic TCM profile, in NCICT the selection of a TCM strength value is the only possibility to integrate tube current modulation. This theoretical value activates the generic, non-vendor-specific, TCM model implemented in the dosimetry tool [17]. Therefore, the TCM strength value that results in organ doses that differ minimally from those obtained through patient-specific dose simulations was first defined. Overall, an increase in TCM strength resulted in larger percentage dose differences for all patient BMI categories and for both PET/CT systems. Because a TCM strength of zero relates to a CT scan performed at a fixed tube current value, a TCM strength of 0.25 was suggested for CT examinations acquired with tube current modulation. This was verified by looking at the distribution of the percentage organ dose differences where the difference between the minimal and maximal observed dose difference were then the smallest.

Although unexpected, a decrease in mean organ dose with BMI was observed for CT scans applying tube current modulation and simulated with ImpactMC (Tables 3 and 4). This may be explained by the fact that an increase in BMI does not necessarily mean an increase in the diameter of the patient. The estimated breast doses, however, are nearly the same for all BMI categories, which may be related to the superficial location of the breast together with the applied TCM system. Considering the related standard deviations, for most organs, the observed decreases are rather small. For TCM examinations simulated with NCICT, a rather small increase in organ dose with BMI was found. Most likely, this finding is due to the theoretical TCM model used by the NCICT software [17].

The standard deviations of the mean organ doses normalised to CTDI_vol_ for the whole-body CT examinations are, in general, higher for the calculations with ImpactMC than with NCICT (Tables 3 and 4). This is seen for all BMI categories and for both PET/CT devices. It may be explained by the fact that ImpactMC, for each patient, uses a 3D voxel model created from the patient’s clinical CT images. In this way, the model matches the realistic anatomical features of the patient such as its size, shape and the location of specific radiosensitive organs. Furthermore, patients referred to PET/CT often suffer a pathology that may alter the anatomy or composition of tissues. As recognised by Kopp et al. [23] and Liu et al. [48], these individual physical characteristics may lead to considerable changes in the distribution of the radiation dose and thus in the patient-specific organ dose estimates. Phantom-based dosimetry tools, on the other hand, are unable to take all these individual properties into account. Although NCICT already considers various body and organ sizes, it does not allow for changes in relative organ position, shape or pathologies that may be present in real patients. The NCICT phantoms can thus be seen as a kind of standardised patients. As a result, standard deviations on simulated organ doses are smaller.

When looking at the mean percentage dose differences (Tables 3 and 4), it is seen that, irrespective of the CT scanner and BMI category, NCICT underestimates the thyroid dose. This underestimation in organ dose is also observed for the other organs of interest for underweight patients. Except for the heart, lung and kidney dose on the Siemens PET/CT, this is also found for healthy weight and overweight patients. For patients classified as obese, all other organ doses on the Siemens system are overestimated by NCICT while for the GE system they are only overestimated for the heart and kidneys. However, a variation in percentage organ dose differences has been observed among all patients. Based on the minimum and maximum values found, the observed under- and overestimations in mean organ doses by NCICT does not necessarily mean that this is the case for each patient separately. No correlations were observed with patient length (R^2^ < 0.1) while they were very weak to moderate with patient weight (0.2 < R^2^ < 0.6) and BMI (0.2 < R^2^ < 0.7). Overall, the average percentage difference of NCICT to ImpactMC doses across all organs and BMI categories for examinations performed with TCM was − 5% and − 22% for the Siemens and GE PET/CT, respectively.

The observed under- and overestimations of CT organ doses by NCICT compared to ImpactMC are also seen in the Deming regression curves between the two dose estimation methods (Fig. 5 and Figure S3, Additional File 1). Note that these curves do not distinguish between BMI categories. For the GE PET/CT system, the Deming regression curves are largely located beneath the equality curve or the curve indicating an exact agreement between both dose estimation tools. Only when the regression lines lie closer towards or cross the equality curve, organ doses are, for certain patients, overestimated by NCICT. This is clearly seen for the heart, liver, lungs and kidneys. For the Siemens PET/CT, on the other hand, stronger variations in under- and overestimations of the organ doses are seen. This is also found when looking at the Deming regression curves, which all cross the equality curve at a certain value. The observed variations in organ dose estimations are related to the fact that NCICT does not allow for changes in relative organ position, shape or present pathologies. Besides, NCICT is intrinsically limited to the technical parameters of the reference CT scanner on which the organ dose calculations are based when the CTDI_vol_ is entered directly into the user interface [24].

Bivariate least square or Deming regression analysis requires an estimation of the measurement error. In contrast to the measurement error of ImpactMC which was validated to be 10% [19], little information can be found on the measurement error of NCICT. Therefore, a measurement error of 25% was chosen based on the mean difference between the initial CTDI_vol_ estimated by the NCICT software and the clinical CTDI_vol_ of the CT examination. To assess the sensitivity to an under- or overestimation of this error, Deming regression coefficients were also calculated using a measurement error of 35% and 15%, respectively. Overall, a mean difference of 6–10% and 1–4% was found for, respectively, the *a* and *b* regression coefficient. The smallest differences were observed when the measurement error was underestimated. Nevertheless, the observed deviations are rather small.

Although the 95% confidence intervals of the intraclass correlation coefficients were relatively broad when considering the classification into BMI categories, this not necessarily indicates a poor agreement between both dosimetry tools. As a rule of thumb, around 30 patients should be included in each category [44]. In this study, however, the number of included patients per BMI category was much lower ranging from 1 to 21 patients. Therefore, it is realistic to suggest that the obtained results are biased due to this low number of patients. This may also explain the observed negative lower bound for the ICC of some organs and BMI categories. Negative values namely denote that the within group variance is greater than the between group variance [49]. Disregarding the BMI categories and thus taking all patients together, ICC analysis resulted into a good to excellent agreement between the organ doses estimated with ImpactMC and NCICT for the GE PET/CT. Only for the thyroid a moderate to good level of reliability was found. For the Siemens PET/CT, moderate to good levels of agreement were observed for the breast, heart, liver, lung and kidney doses, while poor to moderate levels were found for the oesophagus and thyroid. Nevertheless, it suggests that NCICT provides a good enough first dose estimate.

In addition, the correlation between the estimated organ doses and the size-specific dose estimate (SSDE) was investigated. This was done for organ doses obtained with ImpactMC and NCICT. Very strong correlations (0.93 < R^2^ < 1.00) were found, irrespective of the organ and CT. For whole-body CT examinations simulated with ImpactMC, the slope of the regression curves is close to one (m ~ 1) for the GE PET/CT, except for the oesophagus and the thyroid. This indicates that, in this case, the SSDE is a relatively good estimate of the organ dose. The dose to the oesophagus and thyroid are, respectively, over- and underestimates by the SSDE with 10% and 30%. For the Siemens PET/CT, on the other hand, the SSDE overestimates the organ dose in most cases (m < 1). A systematic difference of around 20% was observed. This may be related to the TCM system used. Again, the only exception is the thyroid with a difference of around 40%, which may be explained by its superficial location and small size. For the correlations of the NCICT organ doses with the SSDE almost no variation in slope values is observed for the different PET/CT systems. As discussed previously, this is caused by the fact that the organ doses are calculated based on a reference CT scanner. Generally, NCICT organ doses are overestimated by the SSDE. A difference of 10–20% was found for the heart, liver, lungs, kidneys and thyroid, while a difference of 20–30% was seen for the breast and oesophagus. However, note that the organ doses obtained with NCICT are correlated with the SSDE of the real patients and not with the SSDE of the best matching NCICT phantom. This may influence the observed results.

Today, because of regulations concerning quality assurance and retrospective dose calculations, almost all CT, PET/CT or SPECT/CT systems generate a radiation dose structured report (RDSR) that contains all CT scan parameters and dose values including the CTDI_vol_. In most hospitals, these RDSRs are connected to the local dose management system (DMS) where they are connected to the patient CT images from the PACS. Some examples are Dose (Qaelum), DoseWatch (GE) and TeamPlay (Siemens). They allow an easy extraction of all necessary CT scan parameters and dose values needed for organ dose simulations. In addition, some of these DMSs also report the water equivalent diameter of the patient, which is calculated based on the included CT images. This allows us to easily calculate the size-specific dose estimate of each patient and thereby estimate the organ dose based on previously determined regression curves. Regression curves are preferably obtained from patient-specific Monte Carlo dose calculations, although they may require some effort. Especially organ segmentation may be very time consuming. However, as mentioned above, the development of automatic segmentation tools and advances in deep learning may improve this process.

In this study, not all radiosensitive organs of the human body were investigated. This is because the organs of interest were delineated semi-automatically or manually, which is very time consuming. However, this may be solved by the use of the more recently developed automatic segmentation software TotalSegmentator that, nowadays, incorporates a robust segmentation of 117 anatomic structures in CT images [47]. Next, only whole-body dose exposure was studied. All organs of interest were thus located inside the scan region. In this way, the scatter contribution from outside the scan region and overranging effects were avoided. In addition, partially irradiated organs were avoided as well. When for instance the organ doses of a chest CT would be calculated, NCICT overcomes these effects due to the employment of full-body phantoms. However, organ dose calculations with ImpactMC depend on the availability of clinical CT data. This means that the patient CT images are limited to the anatomy-specific scan range. One of our previous studies indicated that, in this case, organ doses are the most accurate for organs entirely in the field of view while for partially irradiated organs the organ dose is overestimated [50]. This overestimation strongly depends on the amount of the organ volume located outside the field of view. Nevertheless, methods exist to get more realistic organ dose estimates such as the use of the ICRP reference masses, correction factors or hybrid computational phantoms to extend the patient’s anatomy. Finally, the number of patients per PET/CT system were subdivided according to their BMI. This resulted, especially for the underweight and obesity categories, into smaller populations which may have an influence on the observed percentage organ dose differences. A larger number and more equal distribution of patients may lead to more accurate dose difference calculations.

Despite the above-mentioned limitations, this study has also some major advantages. Compared to other studies, dose simulations were performed for a, at the time, sufficiently large patient population and two standard PET/CT systems with diagnostic CT capacities from the two most common vendors.

## Conclusions

Compared to the patient-specific Monte Carlo CT dosimetry software ImpactMC, the computational phantom-based dosimetry tool NCICT could provide organ dose estimates within ± 22% for whole-body CT scans acquired with tube current modulation. However, strong variations are observed between patients. Depending on the organ of interest, NCICT under- or overestimates the organ dose. If better accuracies are required, patient-specific Monte Carlo simulations, which include the realistic patient anatomy, are required. Nevertheless, a moderate to excellent agreement between the organ doses obtained from both methods was observed. Finally, organ doses strongly correlate with the size-specific dose estimate. Depending on the organ of interest and the specific CT scanner, the size-specific dose estimate may be a good first estimate of the organ dose.

## Supplementary Information

Additional file1 (PDF 530 KB)

## Data Availability

The authors declare that all data supporting the findings of this study are available within the article.

## Abbreviations

AAPM: American association of physicists in medicine
ATCM: Automatic tube current modulation
BMI: Body mass index
CT: Computed tomography
CTDI_vol_: Volumetric CT dose index
DLP: Dose-length product
DMS: Dose management system
FOV: Field of view
GDPR: General data protection regulation
ICC: Intraclass correlation coefficient
ICRP: International commission on radiological protection
MC: Monte carlo
PACS: Picture archiving and communication system
PET: Positron emission tomography
PET/CT: Positron emission tomography/computed tomography
RDSR: Radiation dose structured report
ROI: Region of interest
SD: Standard deviation
SPECT/CT: Single photon emission computed tomography/computed tomography
SSDE: Size-specific dose estimate
TCM: Tube current modulation
TLD: Thermoluminescent dosimeter
WB: Whole-body
