# Supplementary material for: Diagnostic CT organ dose estimation in whole-body PET/CT examinations–comparison of a patient-specific monte carlo approach and a computational phantom-based CT dosimetry tool
Source: EJNMMI Phys. 2025 Nov 26;12:102. doi: 10.1186/s40658-025-00811-x (PMC12753603; doi:10.1186/s40658-025-00811-x)
Supplement: Supplementary file 1 — Additional file1 (PDF 530 KB) [file 40658_2025_811_MOESM1_ESM.pdf]

SUPPLEMENTARY INFORMATION

Diagnostic CT organ dose estimation in whole-body PET/CT examinations – Comparison of a patient-specific Monte Carlo approach and a computational phantom-based CT dosimetry tool

Gwenny Verfaillie[1], Yves D'Asseler[2,3], Klaus Bacher[1]

[1] Ghent University, Department of Human Structure and Repair, Ghent, Belgium

[2] Ghent University Hospital, Department of Nuclear Medicine, Ghent, Belgium

[3] Ghent University, Department of Diagnostic Sciences, Ghent, Belgium

This supplementary document presents the results that are not shown in the main manuscript.

Accuracy of organ dose estimations

Influence of TCM strength

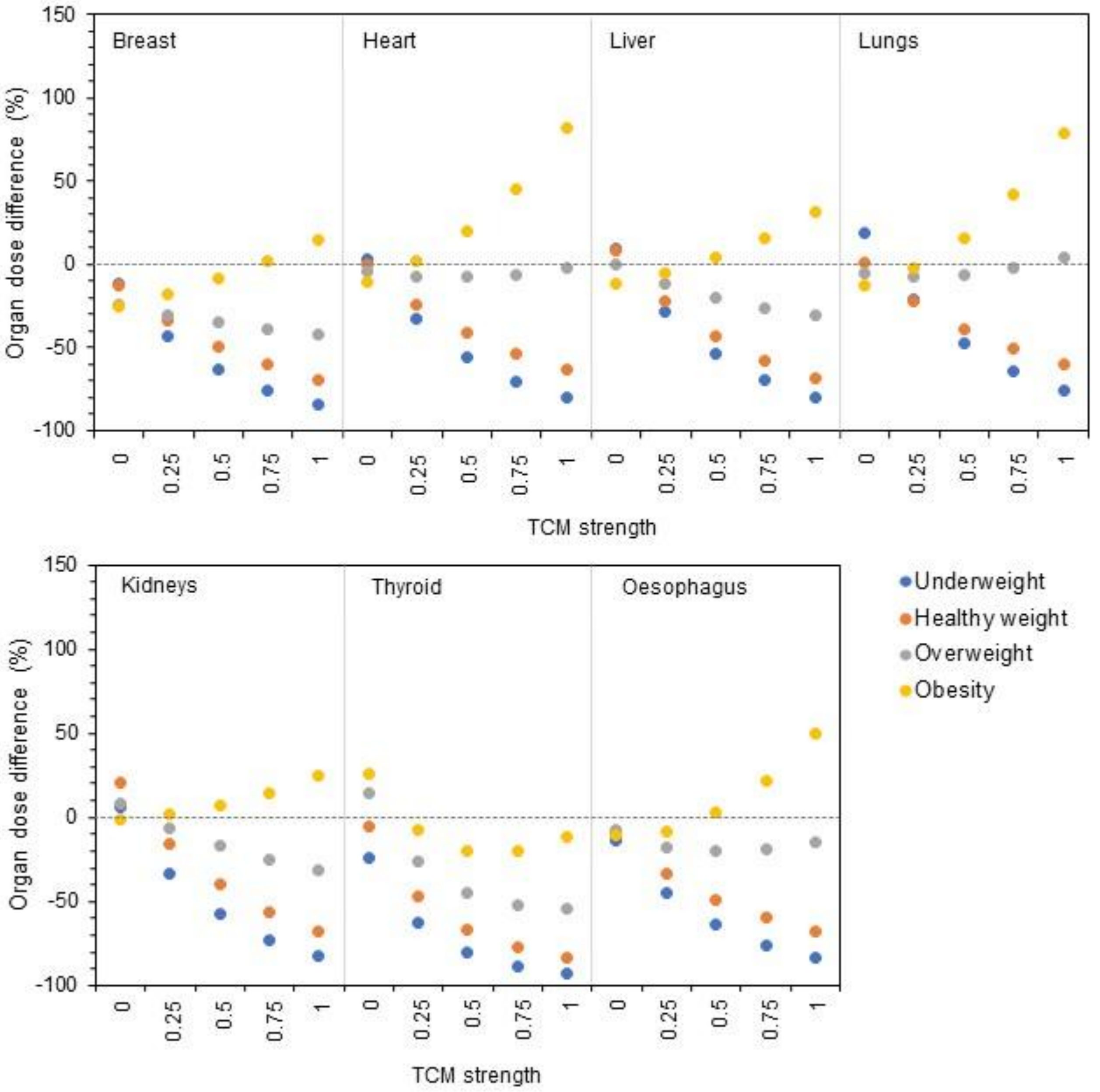

Fig. S1 Percentage difference in mean organ dose, for each BMI category, of a whole-body CT scan at 120 kV on a GE Discovery MI PET/CT simulated with tube current modulation (TCM) using ImpactMC and simulated with different TCM strengths (0, 0.25, 0.5, 0.75 and 1) using NCICT 3.0.

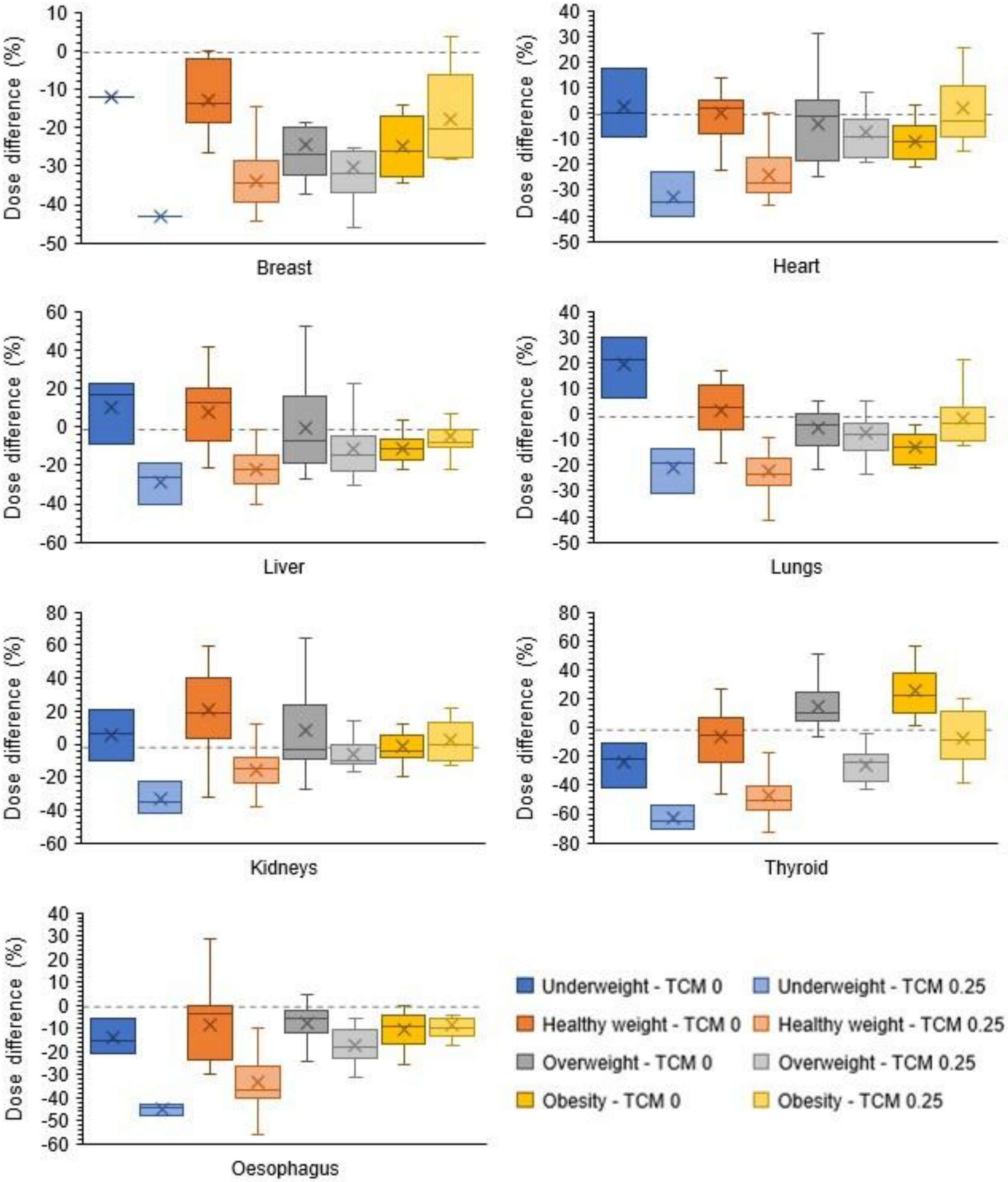

Fig. S2 Distribution of the percentage difference in organ dose, for each BMI category, of a whole-body CT scan on a GE Discovery MI PET/CT simulated with NCICT applying a tube current modulation strength of '0' or '0.25' and simulated with ImpactMC using the clinically applied tube current modulation values.

Organ dose correlations

Correlation between organ doses from ImpactMC and NCICT

Table S1 Two-way model intraclass correlation coefficients (ICC) for single measures and absolute agreement, and their 95% confidence interval, to quantify the agreement between organ doses calculated with ImpactMC and NCICT for diagnostic whole-body CT scans with tube current modulation on a Siemens Biograph mCT Flow PET/CT. A distinction is made between the different BMI categories.

Organ	BMI category	Number of patients	ICC	95% Confidence Interval	
				Lower bound	Upper bound
Breast	Underweight	4	0.82	-0.20	0.99
	Healthy weight	10	0.83	0.45	0.95
	Overweight	6	0.82	0.17	0.97
	Obese	5	0.00	-0.81	0.81
Heart	Underweight	7	0.54	-0.27	0.90
	Healthy weight	20	0.65	0.30	0.85
	Overweight	13	0.70	0.26	0.90
	Obese	10	0.66	0.10	0.90
Liver	Underweight	7	0.26	-0.55	0.82
	Healthy weight	20	0.82	0.60	0.92
	Overweight	13	0.64	0.16	0.87
	Obese	11	0.70	0.21	0.91
Lungs	Underweight	7	0.68	-0.05	0.94
	Healthy weight	20	0.65	0.30	0.84
	Overweight	13	0.63	0.14	0.87
	Obese	11	0.49	-0.13	0.83
Kidneys	Underweight	7	0.00	-0.71	0.71
	Healthy weight	20	0.64	0.28	0.84
	Overweight	13	0.69	0.25	0.89
	Obese	11	0.79	0.39	0.94
Oesophagus	Underweight	7	0.47	-0.35	0.88
	Healthy weight	20	0.48	0.05	0.75
	Overweight	13	0.60	0.10	0.86
	Obese	11	0.28	-0.35	0.74
Thyroid	Underweight	7	0.05	-0.68	0.73
	Healthy weight	19	0.30	-0.17	0.65
	Overweight	12	0.17	-0.42	0.66
	Obese	11	0.72	0.25	0.92

Table S2 Two-way model intraclass correlation coefficients (ICC) for single measures and absolute agreement, and their 95% confidence interval, to quantify the agreement between organ doses calculated with ImpactMC and NCICT for diagnostic whole-body CT scans with tube current modulation on a GE Discovery MI PET/CT. A distinction is made between the different BMI categories. (NA: Not a number)

**Organ**	**BMI category**	**Number of patients**	**ICC**	**95% Confidence Interval**	
				**Lower bound**	**Upper bound**
Breast	Underweight	1	NA	NA	NA
	Healthy weight	11	0.86	0.55	0.96
	Overweight	8	0.78	0.23	0.95
	Obese	5	0.00	-0.81	0.81
Heart	Underweight	3	0.00	-0.95	0.95
	Healthy weight	20	0.86	0.67	0.94
	Overweight	15	0.85	0.62	0.95
	Obese	11	0.20	-0.43	0.70
Liver	Underweight	3	0.00	-0.95	0.95
	Healthy weight	21	0.87	0.70	0.94
	Overweight	15	0.77	0.45	0.92
	Obese	11	0.00	-0.58	0.58
Lungs	Underweight	3	0.58	-0.83	0.99
	Healthy weight	21	0.89	0.75	0.95
	Overweight	15	0.88	0.69	0.96
	Obese	11	0.28	-0.35	0.74
Kidneys	Underweight	3	0.51	-0.85	0.98
	Healthy weight	21	0.81	0.59	0.92
	Overweight	15	0.70	0.31	0.89
	Obese	11	0.01	-0.57	0.58
Oesophagus	Underweight	3	0.82	-0.59	0.99
	Healthy weight	21	0.78	0.54	0.91
	Overweight	15	0.88	0.68	0.96
	Obese	11	0.51	-0.09	0.84
Thyroid	Underweight	3	0.25	-0.92	0.97
	Healthy weight	21	0.66	0.33	0.85
	Overweight	14	0.68	0.26	0.89
	Obese	11	0.29	-0.35	0.74

Table S3 Two-way model intraclass correlation coefficients (ICC) for single measures and absolute agreement, and their 95% confidence interval, to quantify the agreement between organ doses calculated with ImpactMC and NCICT for diagnostic whole-body CT scans with tube current modulation on a Siemens Biograph mCT Flow and a GE Discovery MI PET/CT. No distinction is made between BMI categories.

**PET/CT system**	**Organ**	**Number of patients**	**ICC**	**95% Confidence Interval**	
				**Lower bound**	**Upper bound**
Siemens	Breast	25	0.79	0.59	0.90
	Heart	50	0.62	0.42	0.77
	Liver	50	0.65	0.45	0.78
	Lungs	50	0.77	0.63	0.86
	Kidneys	50	0.73	0.57	0.83
	Oesophagus	50	0.50	0.27	0.68
	Thyroid	49	0.34	0.06	0.56
GE	Breast	25	0.91	0.81	0.96
	Heart	49	0.91	0.84	0.95
	Liver	50	0.92	0.86	0.95
	Lungs	50	0.94	0.90	0.97
	Kidneys	50	0.92	0.86	0.95
	Oesophagus	50	0.90	0.83	0.94
	Thyroid	49	0.70	0.52	0.82

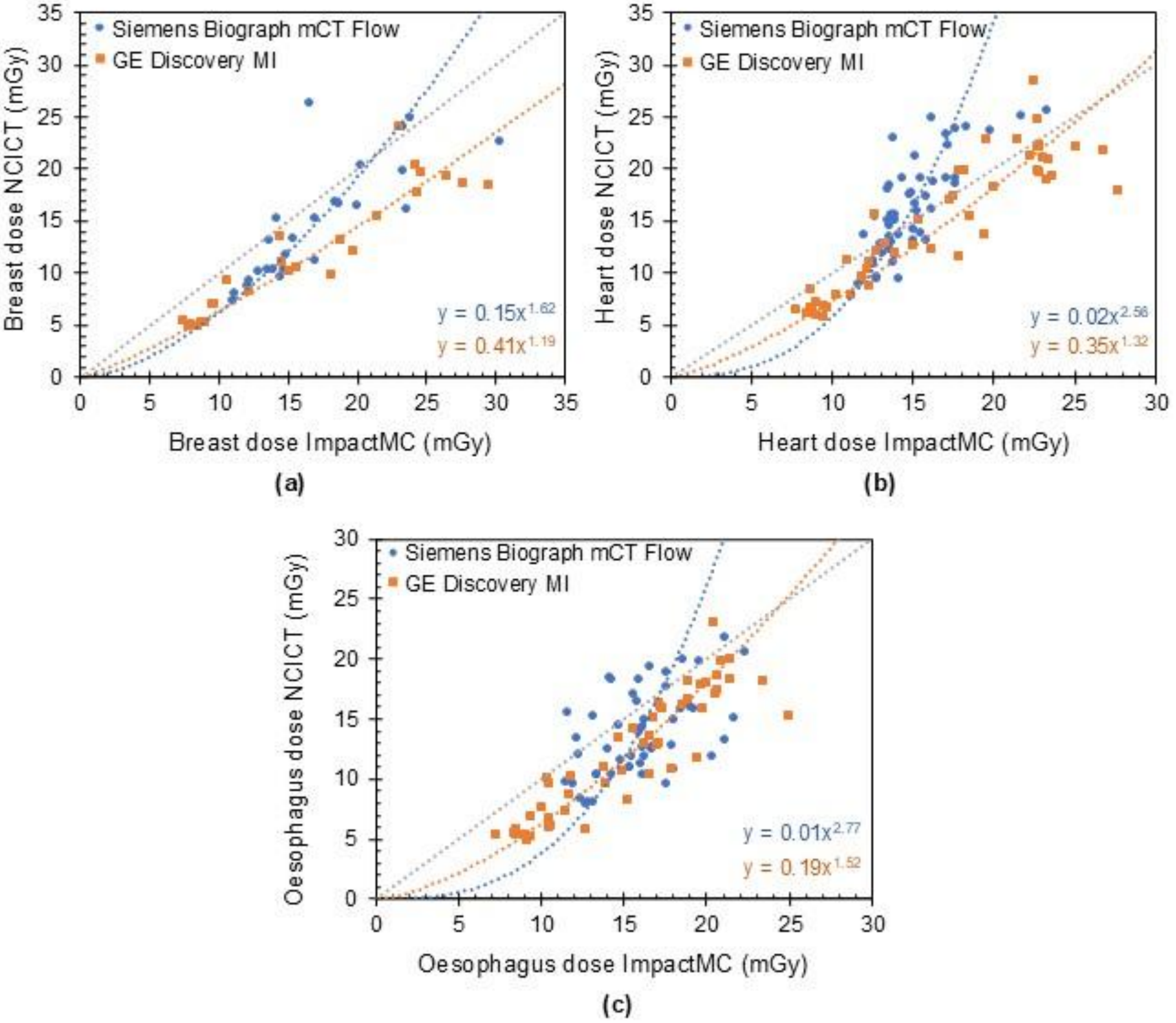

Fig. S3 Relationship between the estimated organ dose calculated with the patient-specific Monte Carlo dosimetry tool ImpactMC and the phantom-based dosimetry software NCICT for a whole-body CT examination on a Siemens Biograph mCT Flow and GE Discovery MI PET/CT acquired with tube current modulation for (a) the breast, (b) the heart and (c) the oesophagus. The added grey dashed line represents the curve that would be followed when both dose calculation methods give the same result.

Correlation of organ doses with SSDE

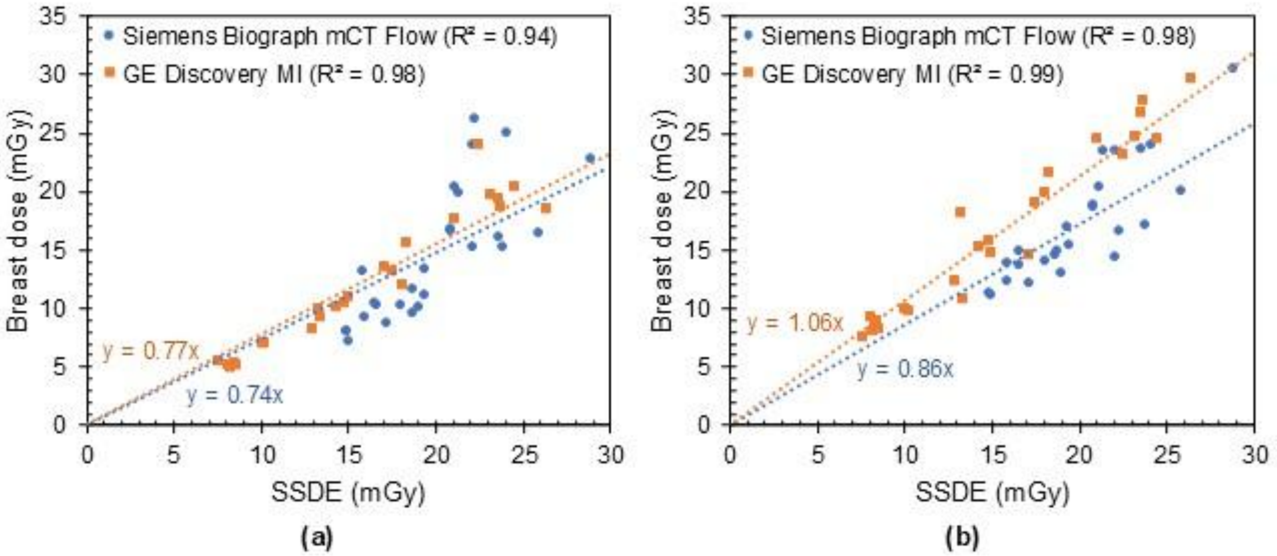

Fig. S4 Estimated breast dose as a function of the size-specific dose estimate (SSDE) for a CT scan at 120 kV with tube current modulation as part of a whole-body PET/CT examination on a Siemens Biograph mCT Flow and GE Discovery MI PET/CT. (a) Organ dose estimation with the phantom-based CT dosimetry tool NCICT and (b) organ dose estimation with the patient-specific CT dosimetry tool ImpactMC.

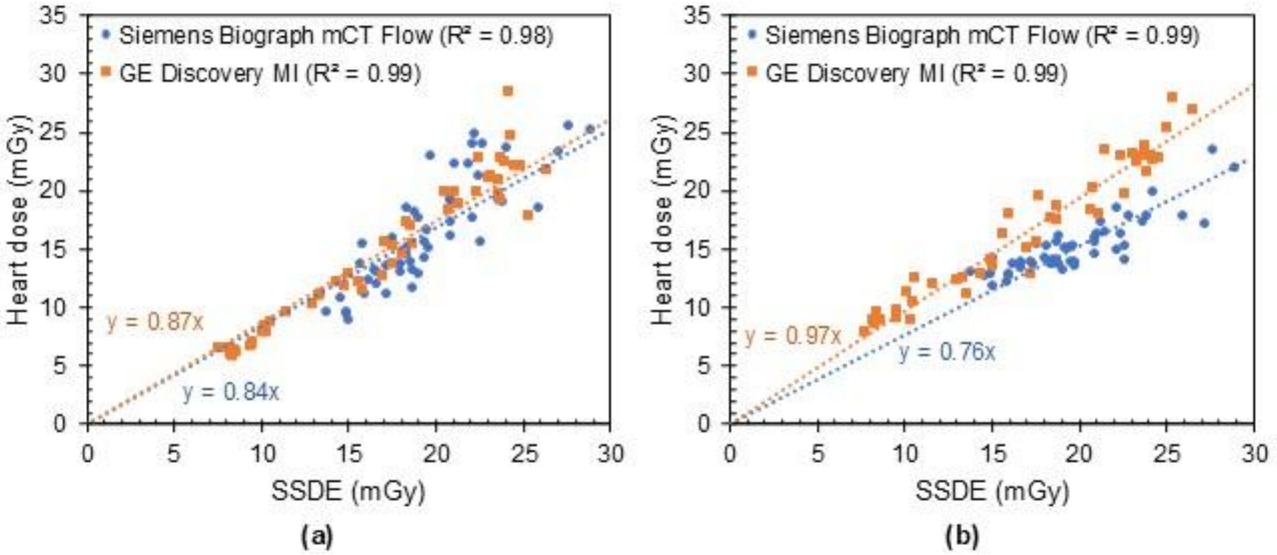

Fig. S5 Estimated heart dose as a function of the size-specific dose estimate (SSDE) for a CT scan at 120 kV with tube current modulation as part of a whole-body PET/CT examination on a Siemens Biograph mCT Flow and GE Discovery MI PET/CT. (a) Organ dose estimation with the phantom-based CT dosimetry tool NCICT and (b) organ dose estimation with the patient-specific CT dosimetry tool ImpactMC.

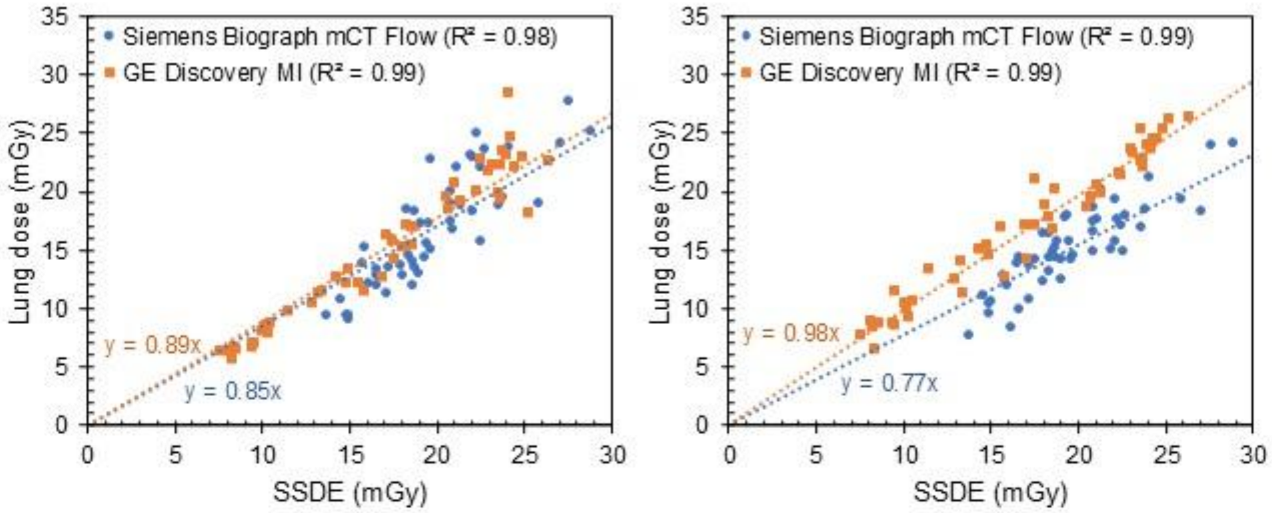

(a) (b)

Fig. S6 Estimated lung dose as a function of the size-specific dose estimate (SSDE) for a CT scan at 120 kV with tube current modulation as part of a whole-body PET/CT examination on a Siemens Biograph mCT Flow and GE Discovery MI PET/CT. (a) Organ dose estimation with the phantom-based CT dosimetry tool NCICT and (b) organ dose estimation with the patient-specific CT dosimetry tool ImpactMC.

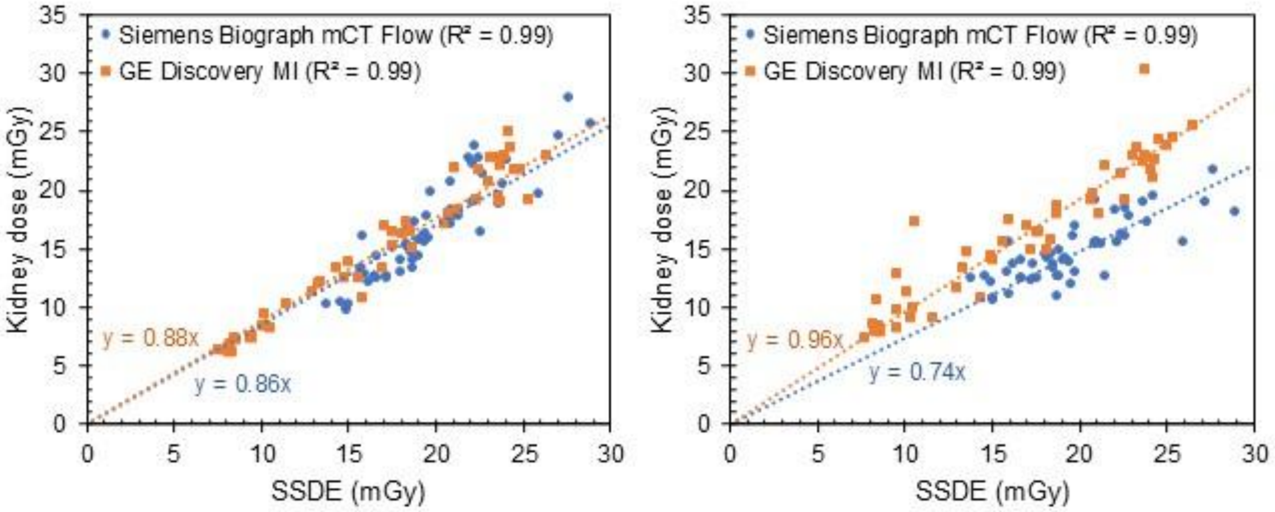

(a) (b)

Fig. S7 Estimated kidney dose as a function of the size-specific dose estimate (SSDE) for a CT scan at 120 kV with tube current modulation as part of a whole-body PET/CT examination on a Siemens Biograph mCT Flow and GE Discovery MI PET/CT. (a) Organ dose estimation with the phantom-based CT dosimetry tool NCICT and (b) organ dose estimation with the patient-specific CT dosimetry tool ImpactMC.

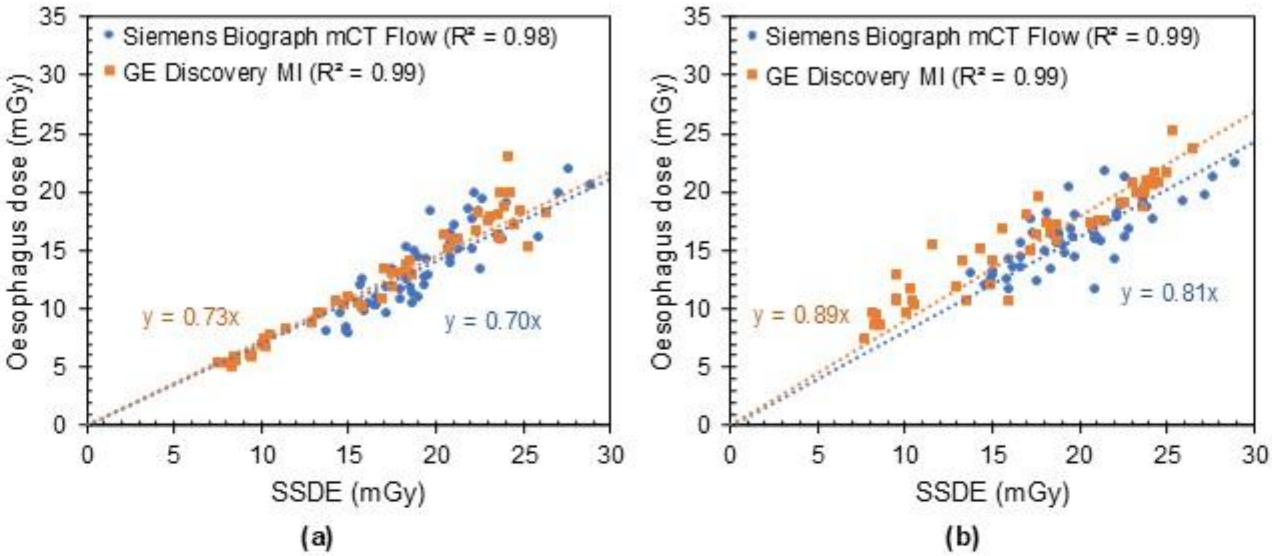

Fig. S8 Estimated oesophagus dose as a function of the size-specific dose estimate (SSDE) for a CT scan at 120 kV with tube current modulation as part of a whole-body PET/CT examination on a Siemens Biograph mCT Flow and GE Discovery MI PET/CT. (a) Organ dose estimation with the phantom-based CT dosimetry tool NCICT and (b) organ dose estimation with the patient-specific CT dosimetry tool ImpactMC.
